# Evaluating the Characteristics and Outcomes of Acute Pharmaceutical Exposure in Children: 5-Year Retrospective Study

**DOI:** 10.2196/66951

**Published:** 2025-06-17

**Authors:** Zhu Yan Duan, Yan Ning Qu, Rui Tang, Jun Ting Liu, Hui Wang, Meng Yi Sheng, Liang Liang Wang, Shuang Liu, Jiao Li, Lin Ying Guo, Si Zheng

**Affiliations:** 1 Emergency Department, Capital Center for Children's Health, Capital Medical University, No. 2 Yabao Road, Chaoyang District, Beijing, 100020, China, 86 13020013651; 2 Capital Institute of Pediatrics, Chinese Academy of Medical Sciences & Peking Union Medical College, Beijing, China; 3 Institute of Medical Information, Chinese Academy of Medical Sciences & Peking Union Medical College, Beijing, China; 4 Child Health Big Data Research Center, Capital Institute of Pediatrics, Beijing, China; 5 Department of Computer Science and Technology, Tsinghua University, Beijing, China

**Keywords:** acute pharmaceutical exposure, children, poisoning, characteristics, outcomes

## Abstract

**Background:**

Acute pharmaceutical exposure in children can lead to severe health outcomes and contribute to the inefficient use of medical resources.

**Objective:**

This study aimed to investigate the clinical characteristics and outcomes of children with acute pharmaceutical exposure to guide the development of preventive strategies and educational initiatives.

**Methods:**

We analyzed real-world data from electronic medical records of children admitted to the emergency department of a pediatric hospital for acute pharmaceutical exposure between January 2019 and December 2023. Clinical data, including laboratory test results, interventions, and outcomes, were collected. We compared different exposure events and conducted logistic regression analysis to identify risk factors for hospitalization.

**Results:**

A total of 653 children were included in the study. The most common drugs involved in exposure were vitamins (149/653, 22.8%), nonsteroidal anti-inflammatory drugs (92/653, 14.1%), and psychiatric drugs (74/653, 11.3%). In total, 74.3% (469/631) of patients with complete clinical manifestation data showed no symptoms after exposure, and 68.1% (445/653) of patients did not require specific therapy. Toxicology screening was performed for 11% (72/653) of the children, and 69.4% (50/72) of these tests were positive. Independent risk factors for hospitalization included multisystem involvement (odds ratio [OR] 4.575, 95% CI 1.709-12.251, *P*=.002), psychiatric drugs (OR 6.280, 95% CI 2.189-18.020, *P*=.001), and intentional poisoning (OR 12.892, 95% CI 2.222-74.796, *P*=.004).

**Conclusions:**

Children with acute pharmaceutical exposure exhibit diverse clinical characteristics and outcomes, with most requiring no specific treatment. However, immediate toxicology screening and clinical intervention are essential for those exhibiting rapidly developing or multisystem symptoms, as well as those with intentional exposure or exposure to known highly toxic substances. Future pediatric health care policies should emphasize safe storage practices and public education on the prevention of pharmaceutical exposure.

## Introduction

Acute poisoning is one of the major public health issues affecting children around the world [1,2]. It can cause severe harm to children and bring immense economic and emotional burdens to their families. As children always lack the ability to metabolize harmful substances, serious physiological and psychological impacts were often observed, even resulting in disability or death [3]. According to the World Health Organization (WHO) report, accidental poisoning is a major contributor to accidental injury-related deaths in children, particularly in limited-income countries, where the mortality from poisoning is more pronounced [4]. Recent studies reported a significant decline in overall childhood poison exposures, but a decrease in poisoning-related fatalities was not observed [5]. Surveys in China also indicate that poisoning events still frequently occurred [5-8]. Meanwhile, acute poisoning has become a common reason for pediatric emergency visits in some regions, accounting for 0.1%‐0.5% of such visits [9,10]. Thus, acute poisoning remains a serious public health concern for pediatrics.

Children, particularly infants and preschoolers, spend the majority of their time at home. Children’s strong curiosity, poor discernment ability, and weak protective awareness make them a high-risk group for pharmaceutical poisoning, often leading to acute poisoning incidents. In addition, unsafe storage of pharmaceuticals or chemicals, inadequate child supervision, nonauthoritative parenting, maternal employment, and lack of family support can also lead to pharmaceutical exposure and poisoning in children [11,12]. For example, Song et al [13] analyzed 586 cases of hospitalized children with acute poisoning and found that the main causes of poisoning included medications (37.7%), pesticides (28.5%), and rodenticides (29.9%). Li et al [14] showed that most poisoning incidents among children are accidental, with 70.4% occurring at home. Accidental poisonings are more common in young children, particularly in those aged 1‐3 years, whereas intentional poisonings are more common among adolescents [15,16]. Furthermore, the clinical manifestations of acute poisoning in children are diverse, and some severe cases presenting consciousness disturbances and circulatory failure can be life-threatening.

Notably, a large proportion of studies focused on pediatric pharmaceutical exposures and related poisoning events have been carried out; however, the epidemiological characteristics and clinical manifestations or outcomes of exposed populations, as well as the toxic substance types and prevention efforts, are often limited to specific research geographical areas, cultural backgrounds, and time periods. Furthermore, with the continuous discovery and development of new drugs, the characteristics of exposed populations are constantly changing, necessitating continuous updates and expansions of the relevant knowledge to meet new challenges for preventing acute poisoning in pediatrics. Additionally, in cases of children with acute pharmaceutical exposure, most parents or caregivers are often unsure whether the drugs are truly dangerous to their children, leading them to immediately take the children to the emergency room. The excessive anxiety and lack of professional knowledge in this population may result in the unnecessary use of pediatric emergency resources. While some exposure incidents indeed require urgent medical intervention, in many cases, the exposure events do not immediately endanger life [17,18].

In light of the aforementioned information, in this study, we retrospectively analyzed the acute pharmaceutical exposure cases from the emergency department of a pediatric hospital located in northern China. By summarizing the epidemiological characteristics and clinical manifestations and outcomes of the patients, this study aims to explore the recent common drugs involved in acute pharmaceutical exposure and the related outcomes, and to propose recommendations for preventing and managing acute pharmaceutical exposure in children.

## Methods

### Sample Collection

This retrospective study included children with acute pharmaceutical exposure who were admitted to the emergency department of the Capital Center for Children’s Health, Capital Medical University between January 1, 2019, and December 31, 2023. All participants were aged 18 years or younger. The inclusion criteria for the patient group were age at assessment between 0 and 18 years, and admission due to pharmaceutical exposure. The exclusion criteria were incomplete records (patients lacking key clinical information such as basic demographic data, type of pharmaceutical substance exposed to, or clinical outcomes), admission due to chronic pharmaceutical exposure, and repeat visits (repeated visit records refer to the follow-up visit records of children with acute pharmaceutical exposure who come to the hospital for further check-ups). Totally, 653 eligible patients were included.

### Data Preprocessing and Variables Extraction

For the patients who met the inclusion criteria, more detailed clinical information was extracted from their anonymized electronic medical records. Specifically, the variables directly extracted from the structured fields of the electronic records included age, gender, residence, admission time, types of pharmaceutical substances (eg, vitamins, psychotropic drugs, and nonsteroidal anti-inflammatory drugs [NSAIDs]), reasons for acute pharmaceutical exposure (intentional, suicidal, or accidental ingestion), exposure location, exposure season, methods of arrival, caregiver’s education level, and clinical outcomes (discharge without treatment, emergency observation, and hospitalization).

The following variables were manually extracted from the unstructured text in the electronic medical records: clinical manifestations (eg, no symptoms, gastrointestinal symptoms, and neurological symptoms), clinical interventions (eg, no specific therapy, gastric lavage, and specific antidotes), and laboratory test results (routine blood test, biochemical test, coagulation function test, and toxicology screening).

The extraction of unstructured data was performed by 4 pediatric emergency physicians with extensive experience. All physicians underwent training on the data extraction content and methods before the process to ensure the accuracy and consistency of the data collection.

All eligible patients were grouped into the following age categories: infancy (<1 year), toddler (≥1 to <3 years), preschool (≥3 to <6 years), school age (≥6 to <12 years), and adolescence (≥12 to <18 years). The pharmaceutical exposure reasons were categorized as intentional and unintentional. The types of drugs involved in acute pharmaceutical exposure were categorized into 13 groups, namely vitamins, NSAIDs (such as ibuprofen and acetaminophen), psychotropic drugs (eg, antidepressant, antiepileptic, and sedative), cardiovascular drugs (eg, antihypertensive and diuretics), antihistamines, respiratory drugs, topical skin drugs, antimicrobial drugs (eg, antibiotic, antiviral, and antifungal drug), endocrine drugs (eg, antidiabetic and hormone), herbal medicine, digestive drugs, immunosuppressants, and others.

After completing the above variable extraction and transformation, 22 patients lacked clinical manifestation data. For laboratory tests, 435 children underwent routine blood tests (C-reactive protein, white blood cells, and platelets), 437 children underwent biochemical tests (liver enzymes, myocardial enzymes, and renal function), 418 children underwent coagulation function tests, and 72 patients underwent toxicology screening. Except for these, all other variables had no missing values. In this study, we did not perform imputation for the variables with missing values, and we only used the actual valid data for each variable for the following data comparison and statistical analysis.

### Statistical Analysis

All analyses were conducted using SPSS (version 26.0; IBM Corp) and Python (version 3.11; Python Software Foundation). The mean level of numerical variables was measured as mean (SD), and differences between different groups were analyzed using 2-tailed *t* tests. Besides, categorical data were measured with frequencies or percentages, and differences between groups were analyzed using the chi-square test, and Fisher exact tests were used when more than 20% of theoretical frequency was less than 5. In addition, univariate and multivariate logistic regression were used and odds ratios (OR) with 95% CIs were calculated to define the risk factors associated with hospitalization for acute pharmaceutical exposure. A *P* value of <.01 was considered statistically significant.

### Ethical Considerations

All methods were performed in accordance with the relevant guidelines and regulations. Since we used anonymized data, and this study did not constitute human subject research, the need for written informed consent was waived by the ethics committee of Children’s Hospital Affiliated to Capital Institute of Pediatrics, due to the retrospective nature of the study (SHERLLM2024029).

## Results

### Characteristics of Children With Acute Pharmaceutical Exposure

**Figure 1.**
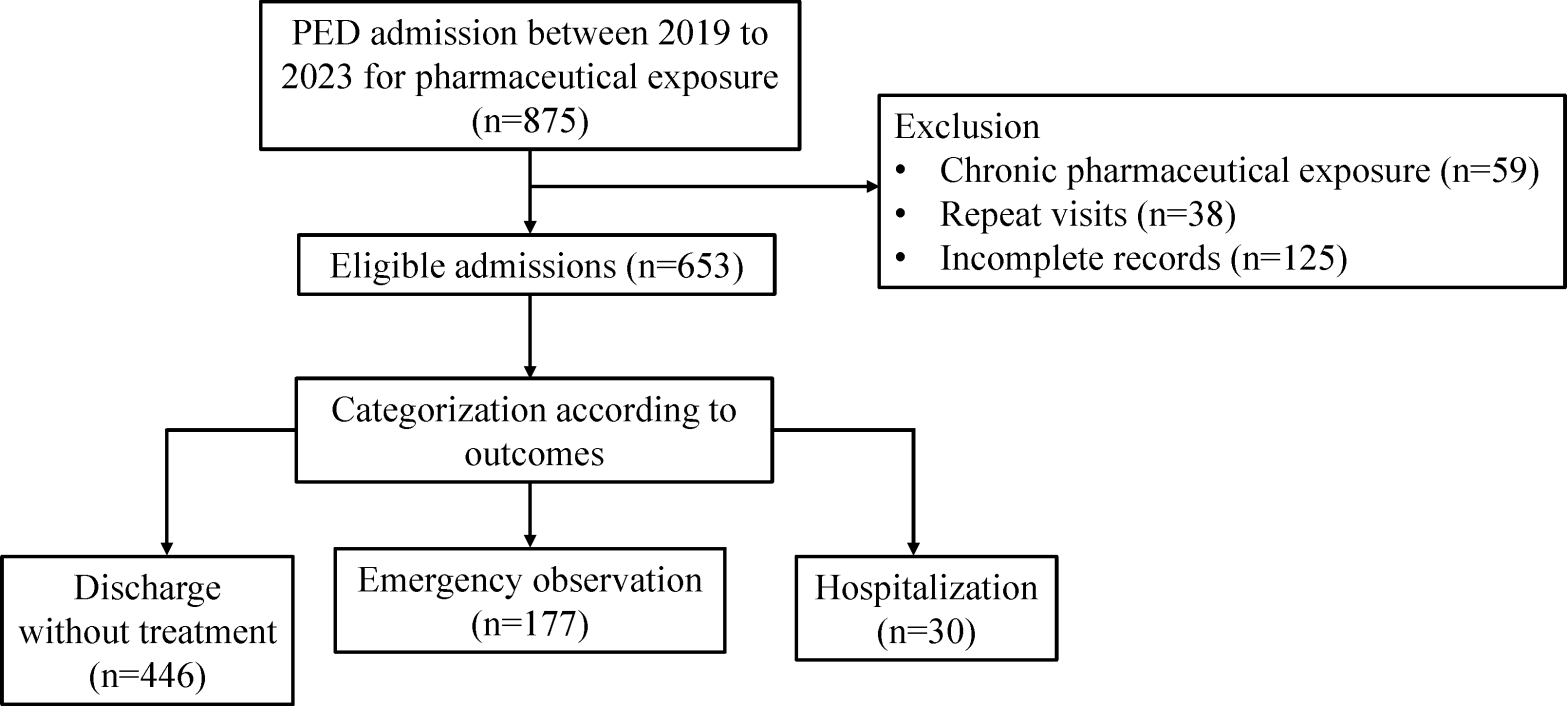
The workflow of sample collection. PED: pediatric emergency department.

From January 1, 2019, to December 31, 2023, 653 adequate pediatric patients with acute pharmaceutical exposure were admitted to our pediatric emergency department (PED; Figure 1). The male-to-female ratio among patients was 1.04:1, with a slightly higher proportion of males. The average age of the patients was 4.7 (SD 4.1) years, with toddlers aged 1‐3 years old accounting for the highest proportion (253/653, 38.7%; Table 1). Analyzing the age distribution by gender, 56.9% (325/571) of the children under 12 years were male and 43.1% (246/571) were female. Among children aged 12 years and older, 11% (9/82) were male and 89% (73/82) were female, which indicated the gender ratio of patients varies across the 2 age groups.

**Table 1.** Characteristics of children with acute pharmaceutical exposure admitted to the pediatric emergency department from 2019 to 2023 (N=653).

Demographic characteristics	Participants, n (%)
Sex	
Male	334 (51.1）
Female	319 (48.9）
Age group	
Infant	47 (7.2）
Toddler	253 (38.7）
Preschool	209 (32）
School age	62 (9.5）
Adolescence	82 (12.6）
Residence	
Urban	574 (87.9）
Rural	79 (12.1）
Exposure place	
Home	608 (93.1）
Others	45 (6.9）
Exposure time	
8 AM to 4 PM	231 (35.4）
4 PM to midnight	356 (54.5）
Midnight to 8 AM	66 (10.1）
Exposure season	
Spring	136 (20.8）
Summer	148 (22.7）
Autumn	175 (26.8）
Winter	194 (29.7）
Exposure reason	
Accidental	575 (88.1）
Intentional	78 (11.9）
Caregiver	
Parents	457 (70）
Grandparents	76 (11.6）
None	102 (15.6）
Others	18 (2.8）
Education level of caregiver	
High school or less	112 (17.1）
Undergraduate or vocational college degree	355 (54.4）
Master’s degree or above	132 (20.2）
Unclear	54 (8.3）

Most pharmaceutical exposure incidents primarily occurred at home, with the peak period mainly occurring between 4 PM and midnight (356/653, 54.5%). Regarding seasonal variations, the number of children visiting the PED for acute pharmaceutical exposure was highest in winter. The primary caregivers at the time of exposure events were parents, and 54.4% (355/653) of the caregivers were college or undergraduates. In addition, analysis of the exposure reasons showed that 11.9% (78/653) were intentional, while the remaining 88.1% (575/653) were accidental. There were 208 poisoning cases (31.9%) resulting from acute pharmaceutical exposure, with 31.3% (65/208) being intentional and the remaining 68.7% (143/208) accidental. In our study, the diagnostic criteria for drug poisoning include the presence of corresponding clinical manifestations, abnormal laboratory results indicating organ damage, or toxicology tests showing drug concentrations reaching toxic levels.

**Figure 2.**
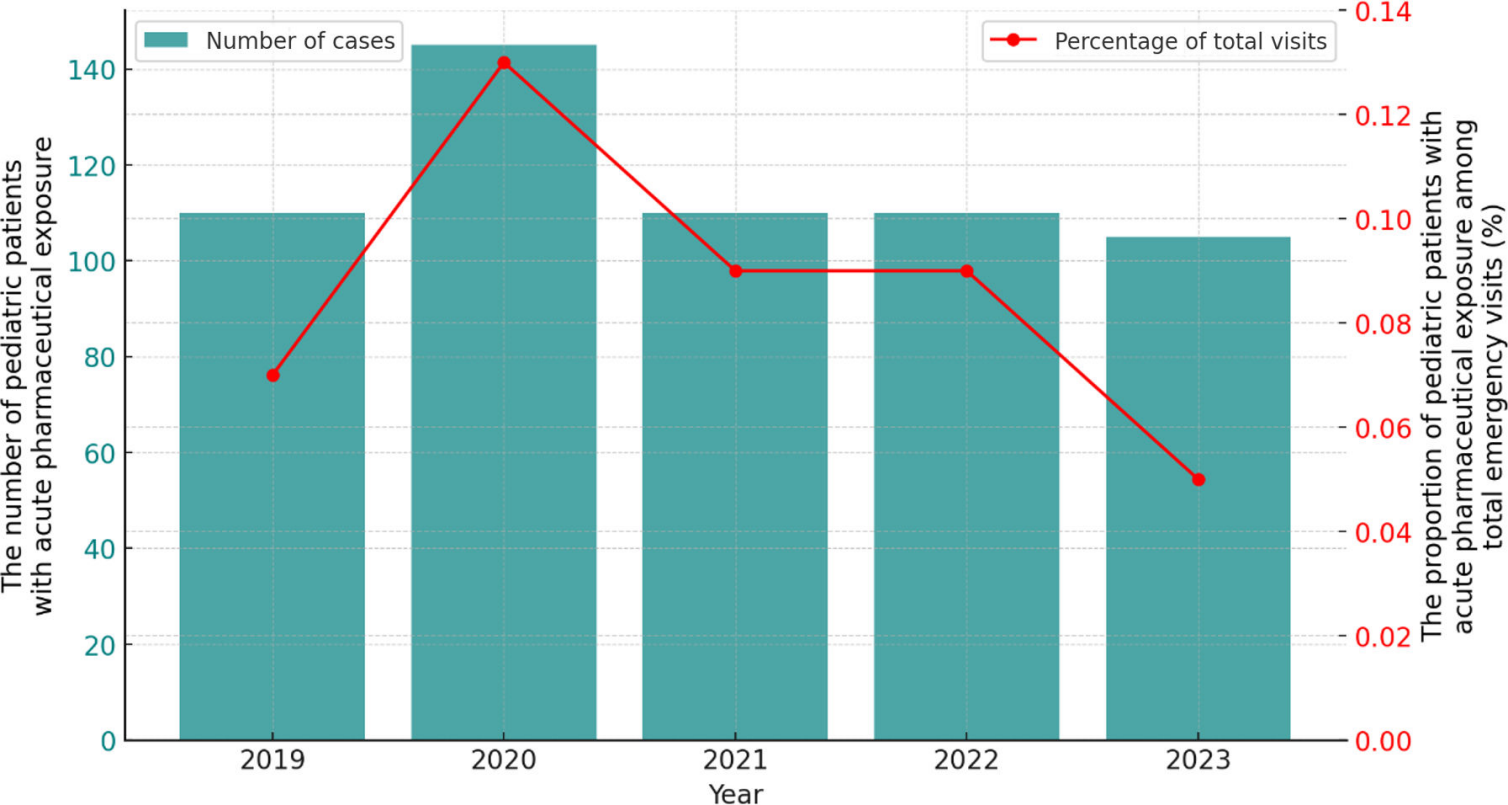
The annual number of pediatric patients with acute pharmaceutical exposure and its proportion in the total annual number of emergency department visits.

In terms of changes over time, the annual number of emergency visits due to acute pharmaceutical exposure fluctuated between 107 and 149 over the past 5 years, with a significant increase in admissions in 2020 (Figure 2). The number of patients in other years did not show significant differences. This increase in the year 2020 may be attributed to prolonged home stays and heightened parental vigilance during the early stages of the pandemic, which led to more admissions related to acute pharmaceutical exposure. The annual proportion of admissions due to acute pharmaceutical exposure ranged from 0.05% to 0.13% of the total number of PED visits.

### Types of Pharmaceutical Substances

**Figure 3.**
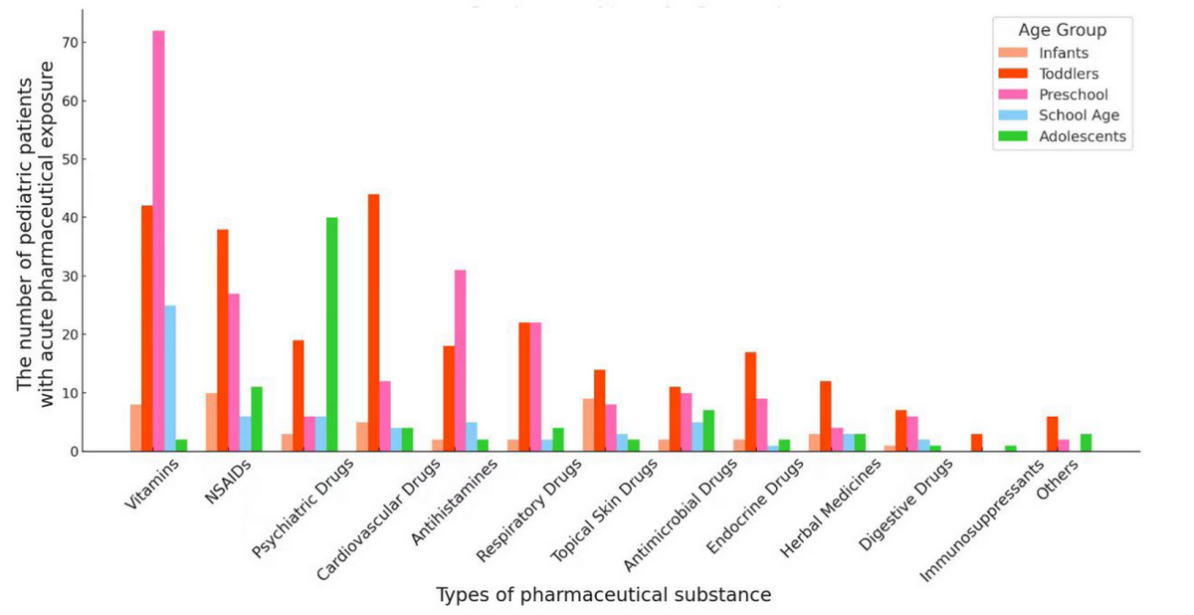
The distribution of pharmaceutical substances across different age groups.

In this study, the drugs involved in acute pharmaceutical exposure were categorized into 13 groups (Figure 3). Totally, vitamins emerged to be the most common in all patients (149/653, 22.8%), followed by NSAIDs (92/653, 14.1%; Table S1 in Multimedia Appendix 1). Notably, the proportion of psychotropic drugs is also high (74/653, 11.3%), of which antidepressants accounted for 64.8% (48/74). Besides, cardiovascular drugs (mainly antihypertensive; 56/69, 81.2%) and endocrine drugs (mainly hypoglycemic agents; 11/31, 35.5%) are also common exposure substances (Table S1 in Multimedia Appendix 1).

Furthermore, the types of pharmaceutical substances varied across different age groups. NSAIDs (10/47, 21.3%) and topical skin drugs (9/47, 19.1%) were the most common substance in infants. While in toddlers, cardiovascular drugs (44/253, 17.4%), vitamins (42/253, 16.6%), and NSAIDs (38/253, 15%) were more prevalent. Preschool and school-age children were most frequently exposed to vitamins, accounting for 34.4% (72/209) and 40.3% (25/62), respectively. In adolescents, psychiatric medications were the most common type (40/82, 48.8%), followed by NSAIDs (11/82, 13.4%; Table S1 in Multimedia Appendix 1). It is worth noting that there were 29 patients (29/653, 4.4%) involved in multiple drug exposures, and 46.4% (12/29) of them were adolescents.

While the pandemic may have slightly influenced the absolute number of admissions (Figure 2), it did not substantially alter the underlying epidemiological trends of pediatric pharmaceutical exposures (Table S2 in Multimedia Appendix 1). Analysis of the types of pharmaceutical substances across different year groups showed temporal fluctuations. For example, between 2020 and 2022, the proportion of neuropsychiatric drug exposure was higher compared with both 2019 and 2023. Conversely, during the pandemic, the proportion of respiratory system drug exposure was lower than before and after the pandemic (5.7% vs 12.1% and 12.1%, respectively). However, the types of pharmaceutical substances involved remained largely consistent across these periods (chi-square test, *P*=.03; Table S2 in Multimedia Appendix 1).

### Clinical Manifestations of Acute Pharmaceutical Exposure

In general, for the 631 patients with complete clinical manifestation data, 74.3% (469/631) did not exhibit obvious symptoms after acute pharmaceutical exposure, while the remaining patients showed one or more system involvements. For instance, 13% (82/631) of the patients showed the involvement of the gastrointestinal system, such as nausea, vomiting, abdominal pain, and diarrhea (Table 2). In total, 6.5% (41/631) of patients exhibited neurological symptoms like dizziness, drowsiness, and even coma in some severe cases; these children are mainly associated with excessive use of antidepressants and sedative overdoses. Cardiovascular symptoms were observed in 4.1% (26/631) of the children and manifested as pallor, sweating, and, in some severe cases, hypotension. In addition, multisystem involvement was noted in 9.5% (60/631) of the cases, and dermatological symptoms were observed in 1.4% (9/631) of the cases.

**Table 2.** Clinical manifestations and the abnormal laboratory test results for children with acute pharmaceutical exposure.

Clinical manifestations (n=631)	Participants, n (%）
No symptom	469 (74.3）
Gastrointestinal symptoms	82 (13）
Neurological symptoms	41 (6.5）
Cardiovascular symptoms	26 (4.1）
Dermatological symptoms	9 (1.4）
Hematological symptoms	1 (0.2）
Multisystem symptoms (>2 systems)	60 (9.5）
Abnormal laboratory test results	
Routine blood test (n=435)	
WBC^a^>12×10^9^/L	58 (13.3）
CRP^b^>8 mg/L	31 (7.1）
WBC＜4×10^9^/L	16 (3.7）
PLT^c^<100×10^9^/L	5 (1.1)
Coagulation function test (n=418)	
Abnormal coagulation index (PT^d^>20 s or APTT^e^>50 s or FIB^f^<2 g/L)	32 (7.7)
Biochemical test (n=437)	
CKMB^g^>3.6 ng/mL	55 (12.6)
ALT^h^>40 U/L	22 (5.0）
Abnormal renal function (urea>7.1 mmol/L or CREA^i^>106 µmol/L)	7 (1.6）

a WBC: white blood cell.

b CRP: C-reactive protein.

c PLT: platelet.

d PT: prothrombin time.

e APTT: activated partial thromboplastin time.

f FIB: fibrinogen.

g CKMB: creatine kinase (CK)-MB.

h ALT: alanine transaminase.

i CREA: creatinine.

In our study, the majority of patients underwent basic laboratory tests, including routine blood tests, biochemistry tests, and coagulation profiles tests. Among these tested populations, as shown in Table 2, the most frequent abnormalities were white blood cell count elevation (58/435, 13.3%) and myocardial enzyme elevation (55/437, 12.6%). Relatively, creatine kinase-MB was frequent in patients exposed to psychiatric drugs (14/49, 28.6%), cardiovascular drugs (10/38, 26.3%), and antimicrobial drugs (6/27, 22.2%; Table S3 in Multimedia Appendix 1). In the antimicrobial drugs exposure group, the proportion of elevated white blood cells and CRP is high (both are 7/26, 26.9%). In addition, in our study, 72 patients underwent toxicology screening, and the total positive rate was 69.4% (50/72); the positive results indicate drug concentrations exceeding therapeutic levels (Table S4 in Multimedia Appendix 1).

### Clinical Interventions and Outcomes of Acute Pharmaceutical Exposure

Before admission, 3.5% (23/653) of the children received emetic treatment, and 1.2% (8/653) of them received drainage from their parents or other caregivers. However, most children with acute pharmaceutical exposure were asymptomatic or showed mild symptoms; 68.3% (446/653) of them did not need specific treatment (discharge after a short observation or without treatment), while the remaining patients received one or more clinical interventions. Gastric lavage was the main treatment strategy for drug removal (145/653, 22.2%), followed by specific antidotes (39/653, 6%) and activated charcoal (38/653, 5.8%). In this study, respiratory support was provided in 1.1% (7/653) of the admitted children when airway protection was necessary due to respiratory or circulatory failure. Extracorporeal techniques like continuous renal replacement therapy, hemoperfusion, or plasma exchange were used in 0.8% (5/653) of the patients (Table 3).

**Table 3.** Clinical interventions and outcomes of patients with acute pharmaceutical exposure (n=653).

Clinical interventions	Participants, n (%）
No specific therapy	445 (68.1）
Gastric lavage	145 (22.2）
Specific antidotes	39 (6.0）
Activated charcoal	38 (5.8）
Induction of vomiting	23 (3.5）
Catharsis	8 (1.2）
Respiratory support	7 (1.1）
CRRT^a^/hemoperfusion/plasma exchange	5 (0.8）
Outcomes	
Discharge without treatment (<6 hours)	446 (68.3）
Emergency observation (≥6 hours)	177 (27.1）
Hospitalization	30 (4.6）

a CRRT: continuous renal replacement therapy.

### Comparison Between Intentional and Unintentional Acute Pharmaceutical Exposures

In this study, 11.9% (78/653) of the children experienced intentional acute drug exposure, with an average age of 13.6 years (Table 4). Of these, 88.5% (69/78) were female and 94.9% (74/78) had a history of depression. Psychotropic drugs were the most common pharmaceutical substances involved in exposure (42/78, 53.2%). In contrast, the average age for unintentional exposures was 3.3 years, with a relatively balanced gender ratio. For unintentional exposures, vitamins were the most common pharmaceutical substances involved in exposure (125/575, 21.7%). In addition, among patients with intentional exposure, 26 underwent toxicology screening, with a positive rate of 92.3% (24/26), which is significantly higher than the unintentional exposure groups (26/46, 56.5%; *P*=.004). Intentional exposures resulted in more severe clinical outcomes, with a significantly higher hospitalization rate compared with unintentional exposures (20.5% vs 2.4%, *P*=1.486×10^−8^). Detailed information is provided in Table 4.

**Table 4.** Comparison of patients with intentional and unintentional acute pharmaceutical drug exposure.

Characteristics	Intentional exposure (n=78)	Unintentional exposure (n=575)	*P* value^a^
Age (years), mean (SD)	13.6 (1.6)	3.3 (2.2)	—^b^
Gender, n (%)			<.001
Male	9 (11.5)	305 (53)	
Female	69 (88.5)	270 (47)	
Patients who were depressed, n (%)	74 (94.9)	0 (0)	
Toxicology screening^c^, n (%)			.004
Positive	24 (92.3)	26 (56.5)	
Negative	2 (7.7)	20 (43.5)	
Multisystem involvement, n (%)			<.001
Yes	22 (28.2)	38 (6.9)	
No	56 (71.8)	515 (93.1)	
Hospitalization, n (%)			<.001
Yes	16 (20.5)	14 (2.4)	
No	62 (79.5)	561 (97.6)	

a *P* value was calculated with chi-square test, except for comparisons between different hospitalization outcomes, which were calculated with Fisher exact test.

b Not applicable.

c The total number of patients with toxicology screening results was 72.

### Factors Influencing Hospitalization for Acute Pharmaceutical Exposure

Univariate analysis indicated that gender, ages 12‐18 years, multisystem involvement, psychiatric drugs, multiple drug exposures, and intentional exposure were significant risk factors for hospitalization (*P*<.01; Table 5). The hospitalization rate was significantly higher for females (25/319, 7.8%) compared with males (5/334, 1.5%), patients over 12 years (20/82, 24.4%) compared with those under 12 years (10/571, 1.8%), patients with multisystem symptoms (19/60, 31.7%) compared with those without (11/593, 1.9%), psychiatric drug exposures (21/74, 28.4%) compared with other drugs (9/579, 1.6%), multiple drug exposures (7/29, 24.1%) compared with single drug exposures (23/624, 3.7%), and intentional poisoning (16/78, 20.5%) compared with accidental ingestion (14/575, 2.4%). For the variables that were significant in univariate analysis, further multivariate analysis was conducted. Further multivariate analysis showed that multisystem symptoms, psychiatric drug, and intentional exposure were independent risk factors for hospitalization (*P*<.01; Table 5).

**Table 5.** Univariate and multivariate logistic regression defined significant risk factors related with hospitalization for children with acute pharmaceutical exposure.

	Hospitalization, n		Univariate analysis^a^		Multivariate analysis	
	No	Yes	*P* value	Odds ratio (95% CI)	*P* value	Odds ratio (95% CI)
Sex			.001	5.614 (2.122‐14.855)	.42	1.709 (0.467‐6.258)
Female	294	25				
Male	329	5				
Age group (years)			<.001	18.065 (8.091‐40.330)	.25	0.344 (0.056‐2.104)
12‐18	62	20				
<12	561	10				
Multisystem involvement			<.001	23.592 (10.522‐52.897)	.002	4.575 (1.709‐12.251)
Yes	41	19				
No	560	11				
Types of pharmaceutical substances			<.001	25.050 (10.923‐57.451)	.001	6.280 (2.189‐18.020)
Psychiatric drug	53	21				
Others	570	9				
Number of drugs			<.001	8.300 (3.220‐21.398)	.10	2.825 (0.805‐9.908)
Multiple drugs	22	7				
Single drug	601	23				
Intentional exposure			<.001	27.680 (11.439‐66.980)	.004	12.892 (2.222‐74.796)
Yes	62	16				
No	561	14				

a For univariate analysis, only variables with *P* value <.05 were shown in the table.

## Discussion

### Principal Findings

This study provides a retrospective analysis of 653 cases with acute pharmaceutical exposure from 2019 to 2023. Our results suggest that toddler and preschool-age groups are more likely to be exposed to drugs. This finding also aligns with Soave et al’s [19] study, which reported an average age of 30 months for poisoned children, highlighting the vulnerability of this age group to drug exposure. Following exposure, most children exhibited no symptoms and did not require specific therapy. The most common manifestations were gastrointestinal symptoms, along with elevated white blood cell counts, which were the most frequent abnormal laboratory test results. We also found that multisystem symptoms, psychiatric drugs, and intentional poisoning were associated with hospitalization after acute pediatric pharmaceutical exposure. Notably, female adolescents with mental health issues were more likely to experience intentional exposure, and positive toxicology screening rate, multisystem involvement, and hospitalization incidence were higher in intentional poisoning group [20].

Regarding regional distribution, this study reveals that 87.9% of the acute pharmaceutical exposure events occurred in urban areas, which could be attributed to the hospital’s location. Research from different regions could show significant differences in the incidence and main types of poisoning in children. For example, studies in Zhejiang [7] and southwestern China [14] found poisoning was more common among rural children, with pesticides and insecticides being as the primary toxic substances. This difference may be due to the higher prevalence of agricultural activities and more frequent chemical use in these areas. In addition, 93.1% of pharmaceutical exposure in our study occurred at home, consistent with previous studies [21,22]. Urban families, especially those with young children, often stockpile medications for common illnesses, inadvertently increasing the likelihood of children accessing these drugs. This highlights the importance of safe storage practices at home. In addition, the peak times for exposure incidents were between 4 PM and midnight, likely due to lapses in parental supervision during this period, as children are more likely to access medications after school. Parents, who were the primary caregivers in 57.2% of cases, generally had a college or university degree, reflecting the central role of parents in our studied area. Despite this, the frequency of pediatric drug exposure suggests a need for greater efforts in educating families about medication safety and preventive measures.

Various studies have indicated that psychiatric medications are commonly associated with acute poisoning in children. For instance, Vilaça et al [23] in Brazil found that anxiolytics (mainly benzodiazepines) were the primary drugs involved in poisoning, followed by analgesics. Anderson et al [24] in the United Kingdom reported that benzodiazepines accounted for 19% of poisoning cases, while Santiago et al [25] in Spain also found that benzodiazepines and detergents were the most commonly involved toxicants. These findings contrast with our results, where vitamins were the most common type of drug exposure, particularly among toddler, preschool and school-aged children. This discrepancy may be due to the fact that caregivers in our study population often keep vitamin supplements at home. However, these vitamins, often presented in candy or liquid form, can attract children and increase the risk of accidental ingestion. Caregivers tend to underestimate the risks associated with vitamins, considering them “harmless,” even though high doses of certain vitamins (eg, vitamins A and D) can cause severe toxic reactions. Therefore, it is crucial to educate caregivers on the safe storage and proper use of vitamins. In the infant group, NSAID exposure was relatively high, aligns with other studies [23]. NSAIDs (eg, ibuprofen and acetaminophen) are commonly used to relieve fever and mild to moderate pain, and fever is prevalent among infants, leading to NSAIDs being a staple in homes. This increases the risk of accidental ingestion, with a significant proportion of infant exposure resulting from caregivers misusing doses, leading to overdose and poisoning incidents. In addition, our study also found that exposure events involving psychiatric medications, primarily antidepressants, were most common among adolescents.

It is also worth noting that 11.9% of the children were intentionally exposed to drugs, primarily among adolescents, with females accounting for 89% of this group. Intentional exposure typically involves larger doses or multiple drugs, resulting in more severe symptoms. Similar to previous studies [26,27], the rate of multiple drug ingestion was higher in adolescents, often linked to suicidal or self-harm intentions, reflecting the vulnerability of this age group to mental health issues. Studies from the United States, Singapore, Taiwan, Sri Lanka, and other regions [28-31] also indicate that adolescent girls face greater psychological and social pressures, making them more prone to emotional fluctuations and self-harm behaviors. Addressing intentional drug exposure requires strengthened psychological interventions and social support, especially mental health screening and intervention, to prevent poisoning incidents. Collaboration among schools, families, and society is crucial to help adolescents establish a healthy mental state. Furthermore, multivariate regression analysis identified psychiatric drug exposure, multisystem involvement, and intentional poisoning as key factors influencing hospitalization in children with acute drug exposure. Werner and Platt’s study [32] in Brazil also showed that intentionality was associated with hospitalization. For these high-risk children, early identification, close monitoring, and proactive intervention are essential to optimize treatment and reduce the severity and mortality of poisoning. More research is needed to refine and validate these factors to guide clinical practice and improve early management of acute drug poisoning.

In our study, most children exposed to drugs showed no clinical symptoms, indicating that many ingested drugs were in small doses or of lower toxicity. Symptoms, when present, primarily affected the digestive and nervous systems, followed by the circulatory system, with multisystem involvement in 9.5% of cases. This is consistent with a study in Chongqing, China [13]. Clinical manifestations in children are closely related to the type and dose of ingested drugs. Higher rates of involvement in the digestive, nervous, and circulatory systems are mainly due to the accidental ingestion of NSAIDs, psychiatric medications, and antihypertensive drugs, which are more likely to cause significant clinical symptoms. Laboratory tests are crucial for diagnosing and guiding treatment in children with acute pharmaceutical exposure. In this study, the majority of the children underwent laboratory tests, with common abnormal results including elevated white blood cell counts and myocardial enzymes. The former likely reflects an inflammatory response rather than an adverse drug reaction. Elevated myocardial enzymes suggest potential myocardial damage from acute drug poisoning, particularly with nervous system drugs, antihypertensive drugs, and NSAIDs [33,34], indicating these drugs have a higher likelihood of causing myocardial damage. In addition, some children exhibited coagulation abnormalities, while liver enzyme and kidney function abnormalities were relatively low, suggesting a lower risk of liver and kidney damage in this study. The study also found that almost all asymptomatic children had normal laboratory results, consistent with Wang et al’s [35] study in the United States, which found no positive results in extensive screening and electrocardiography tests for asymptomatic children aged 12 years or younger. Therefore, the necessity of these tests in asymptomatic children with accidental drug overdoses remains debatable.

Clinical interventions for suspected drug poisoning include detoxification; specific antidotes; and, in severe cases, extracorporeal techniques such as continuous renal replacement therapy, hemoperfusion, and plasma exchange. However, only 0.8% of patients required these advanced treatments. Managing acute drug exposure in children necessitates a comprehensive approach, considering factors such as toxin type, ingestion time, dosage, clinical symptoms, and laboratory results. Toxicology screening, performed in 11% of cases with a 69.4% positive rate, is essential for identifying toxins and assessing the severity of exposure. Studies have shown that toxicology results can optimize treatment plans, preventing both overtreatment and undertreatment [36]. Despite this, 68.1% of children did not require specific treatment, suggesting a potential overuse of emergency services. Improving parental education and raising public awareness of the risks associated with drug exposure could help reduce unnecessary visits to the emergency department and optimize resource allocation. Most children with mild or no symptoms can be safely managed at home with proper guidance, while moderate to severe cases require medical observation or hospitalization. The average cost of a pediatric emergency department visit, including laboratory tests, ranges from ¥1000 to ¥3000 (approximately US $140-US $420), placing a financial burden on families and straining hospital resources. This inefficiency may also contribute to delays in care for critically ill patients.

Generally, preventing accidental drug exposure in children is crucial for reducing poisoning incidents, easing emergency department pressure, and optimizing pediatric medical resource use. Families should store medications out of children’s reach, preferably in locked cabinets, and avoid placing them in common areas like kitchens and bedrooms. Parents should also refrain from taking or handling medications in front of children to prevent curiosity and mimicry [37]. In addition, caregivers should be educated on recognizing early symptoms of poisoning and proper emergency response methods. Child-resistant packaging should be used to minimize the risk of accidental openings [38]. Public health departments should promote medication safety through community activities, brochures, and media campaigns. Schools should incorporate medication safety into health education curricula to teach children about proper medication use and potential dangers from an early age [39]. A comprehensive, multifaceted approach is essential to reducing unnecessary pediatric poisonings from pharmaceutical exposure.

### Limitations

This study has several limitations. First, its single-center design limits the generalizability of the results, and multicenter studies are needed to validate these findings. Second, additional follow-up data are required to explore the long-term effects of pharmaceutical toxic exposures in children. Third, factors such as family health issues, dietary habits, and the pandemic-related lockdown measures were not considered in detail. The increased exposure to neuropsychiatric drugs may be linked to the significant rise in psychological stress faced by children and adolescents during the COVID-19 pandemic. Factors such as prolonged isolation, internet-based learning, and reduced social interactions likely contributed to a surge in anxiety, depression, and other mental health issues, which, in turn, increased the use and exposure to neuropsychiatric drugs. The preventive measures implemented during the pandemic, such as mask-wearing, social distancing, and limited outdoor activities, may have effectively reduced the transmission of respiratory infections, resulting in a decreased incidence of pediatric respiratory diseases and a significant reduction in respiratory drug exposure. Despite these limitations, our study provides valuable insights into the occurrence and outcomes of acute pharmaceutical exposures in children in recent years. Future research with larger sample sizes, longer time spans, and more follow-up data will allow for more comprehensive comparative analysis.

### Conclusions

In conclusion, our study offers valuable insights into the epidemiological characteristics and clinical presentations of children with acute drug exposure. It also emphasizes the importance of ongoing education and preventive measures to reduce the risk of drug poisoning in children and optimize the use of emergency medical resources.

## Supplementary material

10.2196/66951 Multimedia Appendix 1 Supplementary materials on different types of pharmaceutical exposure in different age groups, the distribution of pharmaceutical substances in different year groups, abnormal laboratory test results in different pharmaceutical exposure groups, and positive toxicology screening results in different pharmaceutical exposure groups.

## Abbreviations

NSAID: nonsteroidal anti-inflammatory drug
OR: odds ratio
PED: pediatric emergency department
WHO: World Health Organization
