# Supplementary material for: Evaluating the Characteristics and Outcomes of Acute Pharmaceutical Exposure in Children: 5-Year Retrospective Study
Source: JMIR Pediatr Parent. 2025 Jun 17;8:e66951. doi: 10.2196/66951 (PMC12187026; doi:10.2196/66951)
Supplement: Multimedia Appendix 1 [file pediatrics-v8-e66951-s001.docx]

**Table S1.** The distribution of patients with different types of pharmaceutical exposure in different age groups.

| **Types of pharmaceutical substance** | **Vitamins** | **NSAIDs** | **Psychiatric drugs** | **Cardiovascular drugs** | **Antihistamines** | **Respiratory drugs** | **Topical skin drugs** | **Antimicrobial drugs** | **Endocrine drugs** | **Herbal medicines** | **Digestive drugs** | **Immunosuppressants** | **Others** |
| --- | --- | --- | --- | --- | --- | --- | --- | --- | --- | --- | --- | --- | --- |
| **Infancy, N(%)** | 8  (17%) | 10 (21.3%) | 3  (8.4%) | 5  (10.6%) | 2  (4.3%) | 2  (4.2%) | 9 (19.1%) | 2  (4.3%) | 2  (4.3%) | 3  (6.4%) | 1 (2.1%) | 0  (0%) | 0  (0%) |
| **Toddler, N(%)** | 42  (16.6%) | 38 (15.0%) | 19  (7.5%) | 44  (17.4%) | 18  (7.1%) | 22  (8.7%) | 14 (5.5%) | 11  (4.3%) | 17 (6.8%) | 12 (4.7%) | 7 (2.8%) | 3  (1.2%) | 6  (2.4%) |
| **Preschool, N(%)** | 72  (34.4%) | 27 (12.9%) | 6  (2.9%) | 12  (5.7%) | 31  (14.8%) | 22  (10.5%) | 8 (3.8%) | 10  (4.8%) | 9  (4.3%) | 4  (1.9%) | 6 (2.9%) | 0  (0%) | 2  (1.0%) |
| **School age, N(%)** | 25  (40.3%) | 6  (9.7%) | 6  (9.7%) | 4  (6.4%) | 5  (8.1%) | 2  (3.2%) | 3 (4.8%) | 5  (8.1%) | 1  (1.6%) | 3  (4.8%) | 2 (3.2%) | 0  (0%) | 0  (0%) |
| **Adolescence, N(%)** | 2  (2.4%) | 11 (13.4%) | 40 (48.8%) | 4  (4.9%) | 2  (2.4%) | 4  (4.9%) | 2 (2.4%) | 7  (8.5%) | 2  (2.4%) | 3  (3.7%) | 1 (1.2%) | 1  (1.2%) | 3  (3.7%) |
| **Total, N(%)** | 149  (22.8%) | 92  (14.1%) | 74  (11.3%) | 69  (10.6%) | 58  (8.9%) | 52  (8.0%) | 36  (5.5%) | 35  (5.4%) | 31  (4.7%) | 25  (3.8%) | 17  (2.6%) | 4  (0.6%) | 11  (1.7%) |

*.*

**Table S2.** The distribution of pharmaceutical substances in different year groups.

| **Types of pharmaceutical substance** | **pre-pandemic [2019]（n=107）** | **pandemic [2020–2022]**  **（n=422）** | **post-pandemic [2023]（n=124）** |
| --- | --- | --- | --- |
| **Vitamins, N(%)** | 23(21.5%) | 94(22.3%) | 32(25.8%) |
| **NSAIDs, N(%)** | 16(15.0%) | 55(13.0%) | 21(16.9%) |
| **Psychiatric drugs, N(%)** | 6(5.6%) | 59(14.0%) | 9(7.3%) |
| **Cardiovascular drugs, N(%)** | 11(10.3%) | 51(12.1%) | 7(5.6%) |
| **Antihistamines, N(%)** | 13(12.1%) | 33(7.8%) | 12(9.7%) |
| **Respiratory drugs, N(%)** | 13(12.1%) | 24(5.7%) | 15(12.1%) |
| **Topical skin drugs, N(%)** | 5(4.7%) | 27(6.4%) | 4(3.2%) |
| **Antimicrobial drugs, N(%)** | 4(3.7%) | 21(5.0%) | 10(8.1%) |
| **Endocrine drugs, N(%)** | 3(2.8%) | 22(5.2%) | 6(4.8%) |
| **Herbal medicines, N(%)** | 7(6.6%) | 15(3.6%) | 3(2.4%) |
| **Digestive drugs, N(%)** | 2(1.9%) | 14(3.3%) | 1(0.9%) |
| **Immunosuppressants** | 1(0.9%) | 3(0.7%) | 0(0.0%) |
| **Others, N(%)** | 3(2.8%) | 4(0.9%) | 4(3.2%) |

**Table S3.** The distribution of patients with abnormal laboratory test results in different pharmaceutical exposure groups^*^.

| **Abnormal laboratory test results** | **Vitamins** | **NSAIDs** | **Antihistamines** | **Cardiovascular drugs** | **Psychiatric drugs** | **Topical skin drugs** | **Antimicrobial drugs** | **Endocrine drugs** | **Herbal medicines** | **Respiratory drugs** | **Digestive drugs** | **Immunosuppressants** | **Others** |
| --- | --- | --- | --- | --- | --- | --- | --- | --- | --- | --- | --- | --- | --- |
| **WBC>**  **12×10^9/L, N(%)** | 15/98  (15.3%) | 4/69  (5.8%) | 3/43  (7.0%) | 5/36  (13.9%) | 7/51  (13.7%) | 2/21  (9.5%) | 7/26  (26.9%) | 2/17  (11.8%) | 3/17  (17.6%) | 8/36  (22.2%) | 1/10  (10.0%) | 0/2  (0.0%) | 1/9  (11.1%) |
| **CRP>8mg/L, N(%)** | 5/98  (5.1%) | 7/69  (1.0%) | 1/43  (2.3%) | 2/36  (5.6%) | 1/51  (2.0%) | 0/21  (0.0%) | 7/26  (26.9%) | 1/17  (5.9%) | 2/17  (11.8%) | 5/36  (13.9%) | 0/10  (0.0%) | 0/2  (0.0%) | 0/9  (0.0%) |
| **WBC＜4×10^9/L, N(%)** | 1/98  (1.0%) | 11/69  (15.9%) | 1/43  (2.3%) | 0/36  (0.0%) | 1/51  (2.0%) | 0/21  (0.0%) | 2/26  (7.7%) | 0/17  (0.0%) | 0/17  (0.0%) | 0/36  (0.0%) | 0/10  (0.0%) | 0/2  (0.0%) | 0/9  (0.0%) |
| **PLT＜100×10^9/L, N(%)** | 1/98  (1.0%) | 1/69  (1.4%) | 0/43  (0.0%) | 0/36  (0.0%) | 1/51  (2.0%) | 0/21  (0.0%) | 1/26  (3.8%) | 0/17  (0.0%) | 0/17  (0.0%) | 1/36  (2.8%) | 0/10  (0.0%) | 0/2  (0.0%) | 0/9  (0.0%) |
| **Abnormal coagulation index**  **(PT>20s or APTT>50s or FIB＜2g/L), N(%)** | 9/94  (9.6%) | 6/69  (8.7%) | 3/40  (7.5%) | 3/36  (8.3%) | 4/47  (8.5%) | 1/20  (5.0%) | 2/25  (8.0%) | 1/16  (6.3%) | 0/16  (0.0%) | 2/35  (5.7%) | 0/10  (0.0%) | 0/2  (0.0%) | 1/8  (12.5%) |
| **CKMB>**  **3.6ng/ml, N(%)** | 5/99  (5.1%) | 9/72  (12.5%) | 2/41  (4.9%) | 10/38  (26.3%) | 14/49  (28.6%) | 2/22  (9.1%) | 6/27  (22.2%) | 1/16  (6.3%) | 1/16  (6.3%) | 4/38  (10.5%) | 1/9  (11.1%) | 0/2  (0.0%) | 0/8  (0.0%) |
| **ALT>40U/L, N(%)** | 2/99  (2.0%) | 7/72  (9.7%) | 2/41  (4.9%) | 3/38  (7.9%) | 5/49  (10.2%) | 0/22  (0.0%) | 2/27  (7.4%) | 0/16  (0.0%) | 1/16  (6.3%) | 0/38  (0.0%) | 0/9  (0.0%) | 0/2  (0.0%) | 0/8  (0.0%) |
| **Abnormal renal function**  **(Urea>7.1mmol/L or CREA>106umol/L), N(%)** | 0/99  (0.0%) | 1/72  (1.4%) | 0/41  (0.0%) | 1/38  (2.6%) | 4/49  (8.2%) | 0/22  (0.0%) | 1/27  (3.7%) | 0/16  (0.0%) | 0/16  (0.0%) | 0/38  (0.0%) | 0/9  (0.0%) | 0/2  (0.0%) | 0/8  (0.0%) |

*In total, 435 children underwent routine blood test (C-reactive protein, white blood cells, platelets), 437 children underwent biochemical tests (liver enzymes, myocardial enzymes, renal function), 418 children underwent coagulation function tests. For these children underwent laboratory tests, we calculated the percentage of abnormal test results for each test item in different pharmaceutical exposure groups.

**Table S4.** The distribution of patients with positive toxicology screening results in different pharmaceutical exposure groups.

| **Toxicology screening** | **Vitamins (n=4)** | **NSAIDs (n=11)** | **Antihistamines (n=3)** | **Cardiovascular drugs (n=11)** | **Psychiatric drugs (n=21)** | **Topical skin drugs (n=5)** | **Antimicrobial drugs (n=3)** | **Herbal medicines (n=3)** | **Respiratory drugs (n=5)** | **Endocrine drugs（n=1）** | **Digestive drugs (n=2)** | **Others (n=3)** |
| --- | --- | --- | --- | --- | --- | --- | --- | --- | --- | --- | --- | --- |
| **Positive, N(%)** | 3  (75.0%) | 6  (54.5%) | 2  (66.7%) | 7  (63.6%) | 21  (100%) | 2  (40.0%) | 2  (66.7%) | 0  (0%) | 4  (80%) | 1  （100%） | 1  (50%) | 1  (33.3%) |
